# A Framework for Understanding and Generating Integrated Solutions for Residential Peak Energy Demand

**DOI:** 10.1371/journal.pone.0121195

**Published:** 2015-03-25

**Authors:** Laurie Buys, Desley Vine, Gerard Ledwich, John Bell, Kerrie Mengersen, Peter Morris, Jim Lewis

**Affiliations:** 1 School of Design, Creative Industries Faculty, Queensland University of Technology, Brisbane, Queensland, Australia; 2 School of Electrical Engineer and Computer Science, Science and Engineering Faculty, Queensland University of Technology, Brisbane, Queensland, Australia; 3 School of Chemistry, Physics and Mechanical Engineering, Science and Engineering Faculty, Queensland University of Technology, Brisbane, Queensland, Australia; 4 School of Mathematical and Statistical Sciences, Science and Engineering Faculty, Queensland University of Technology, Brisbane, Queensland, Australia; Tianjin University of Technology, CHINA

## Abstract

Supplying peak energy demand in a cost effective, reliable manner is a critical focus for utilities internationally. Successfully addressing peak energy concerns requires understanding of all the factors that affect electricity demand especially at peak times. This paper is based on past attempts of proposing models designed to aid our understanding of the influences on residential peak energy demand in a systematic and comprehensive way. Our model has been developed through a group model building process as a systems framework of the problem situation to model the complexity within and between systems and indicate how changes in one element might flow on to others. It is comprised of themes (social, technical and change management options) networked together in a way that captures their influence and association with each other and also their influence, association and impact on appliance usage and residential peak energy demand. The real value of the model is in creating awareness, understanding and insight into the complexity of residential peak energy demand and in working with this complexity to identify and integrate the social, technical and change management option themes and their impact on appliance usage and residential energy demand at peak times.

## Introduction

Recently, electricity systems have been examined worldwide for their contribution to environmental issues including climate change, depletion of resources through the continued use of fossil fuels and the consistently rising cost of electricity to customers due to the investment required for upgrading infrastructure to provide power during periods of peak demand [1, 2]. Governments, policy makers and the electricity industry are addressing these issues through measures such as promotion of renewable generation, incentives for energy efficiency and educating customers on energy demand and cost reduction opportunities [1]. However, the adoption rate of energy efficient practice despite the availability and promotion of the incentives for such behaviour would seem to indicate that consumers are making irrational economic decisions [3]. Success in addressing these concerns, therefore, requires understanding of all the technical and social factors that affect electricity demand especially at peak times.

This paper builds upon current knowledge of residential energy consumption and previous efforts to offer models to improve understanding of the effects on residential peak energy demand. This paper introduces and discusses the development of a new conceptual multi-disciplinary complex model to guide analysis of residential energy choices during network peak periods. Past and current research in energy analysis are reviewed. The purpose of this paper is to present a dynamic conceptual framework that enables, in an integrated way, the exploration of the complexity of factors that influence residential consumers’ energy use during peak demand periods.

### Why peak demand is the critical focus

Peak demand for electricity is a critical focus as it has been growing much faster than average demand thus challenging electricity utilities to supply peak demand in a cost effective, reliable manner [4]. Electricity cannot be economically stored in quantities large enough to currently operate a reliable network [5]. This means supply must equal demand at all times as failure to do so results in outages and load shedding causing some customers to lose supply, creating a difficult challenge for the industry. In extreme cases the electricity network could be destabilised, causing a greater loss of supply and widespread blackouts [6, 7]. Such a situation occurred in the United States in August 2003 and affected the lives of 50 million citizens [8]. Electricity industry planning teams are very conservative by nature [9] and design the network for a healthy safety margin in generation, transmission and distribution capacity to match the unpredictable nature of demand due to weather conditions and autonomous use by consumers [10].

Energy distributors based in Queensland Australia forecast that the demand for electricity at peak times, as experienced on hot or humid days, will increase 74% between 2008 and 2020. This contrasts with the total energy consumption in Queensland increasing by 48% in the same period [4]. The Queensland Government estimates that the distributors will spend $1 billion on infrastructure over the next three years to meet demand required during peak times which equates to only 1% of the year [4]. Such rapidly increasing capital investment in electricity provision, use of fossil fuels, damage to the environment and additional cost to the consumer could be delayed or avoided if residential customers voluntarily changed their demand patterns at times of network peaks.

### Exploring the evidence

Over almost forty years, there have been numerous studies from a wide range of disciplinary perspectives (including economics, engineering and sociology, anthropology and psychology) providing different frameworks, theories and designs of interventions to change behaviour of residential electricity customers with none providing a reliably successful predictive tool or intervention [3, 11–14] due to the limited view of considering only a selective set of factors influencing energy use [15]. Previous studies have predominately had an environmental focus, for example [16–24], and so cannot easily be identified as specific peak reduction research. This same research has typically described conservation and efficiency behavioural change as an essential issue. Although the link between conservation, efficiency and peak demand is rarely identified, there is valuable insight within pro-environmental literature to the topic of peak demand reduction and behaviour change.

Research that has specifically addressed residential peak demand have mostly targeted economic variables of peak pricing mechanisms [25–29], pricing and load control [30–32] and price and customer perception [33, 34]. There have been other studies which investigated voluntary load shedding [35], battery storage [36] and the impact of photovoltaics on a building’s peak load [37]. Lutzenhiser [38], however, found that targeting economic variables or psychological variables in isolation can only achieve limited and short-term success in affecting behavioural change.

### The call for a multi-disciplinary approach

There has been no single disciplinary program that has proved reliably successful in understanding energy consuming behaviour or as an intervention in addressing energy conservation [3, 11, 14]. It has been suggested that this failure of numerous theories and interventions is not unexpected given that the supply and demand of electricity exists within a very complex system that has lots of component parts that cannot be reduced to simple explanations or policy approaches [39]. As a result, there has been a growing call for integrated approaches of analysis of residential energy consumption in order to address the multifaceted challenges of energy policy and achieve more realistic and wide-ranging understanding of energy consumption than provided by single disciplinary studies [3, 11, 14, 38]. In a review of home energy consumption research over 30 years, Crosbie [40] found that there needs to be an integration of quantitatively based behaviour modelling with more recent socio-technical qualitative studies. She suggested that research will be most powerful if nuanced and detailed sociological and ethnographic accounts of consumers’ everyday practices are combined with longitudinal and detailed measurements associated with consumer and behaviour work. Other researchers have also advocated the assimilation of socio-technical models with individual behaviour based ones [14]. The lack of progress towards a multi-disciplinary model, however, has been said to be due to past integrated studies dealing more with small scale issues thereby restricting insight at a larger scale [11] and the entrenched theoretical preferences of the various disciplines [14, 38]. Nevertheless, there have been examples of progress towards integrated models including, the behavioral model [41], the model of environmentally significant behavior [42], the multigenic model [43], an agent based integrated framework [11], the energy cultures framework [3] and the three dimensional energy profile framework [44].

Despite evidence of poor performance of physical-technical and economic (PTE) models they continue to dominate energy analysis and influence policy makers [44]. PTE models are based on “the twin technical and economic logics of proven, replicable, science and idealised consumer behaviour” ([45], p. 647). However, the outcomes of human behaviour and natural systems are often uncertain and complex and require a different approach to one strictly based on logic and structure [46, 47]. There are factors other than financial economy which heavily influence residential energy consumption and these include infrastructure and the built environment, technology possibilities and social norms [48]. The human dimension of energy use plays a significant role and yet has been largely overlooked in comparison to PTE models [49]. The everyday processes of energy use involve complex social, cognitive and behavioural processes which are not well understood [11, 16, 38, 48]. While there have been numerous theories, including the diffusion of innovation model, cognitive dissonance and theory of planned behaviour, which have been successfully applied to explain human choices in a wide variety of contexts, these same theories have not been widely used in the energy field [44]. Ongoing improvement of multi-disciplinary approaches is needed to ensure their credibility and to make them a feasible alternative to existing physical-technical and economic based decision making models [11].

Studying and modelling human behaviour sets consumption as an individual behaviour which implies that people make completely sovereign choices, thereby discounting the effect of social expectations such as those relating to proper care of the family, definitions of comfort and healthy living and presumed social expectations of guests [50]. According to anthropologists and sociologists, energy models should consider the social context of individual actions because they believe that human behaviour is social and collective [38]. They have studied people’s everyday practices, (such as bathing, cleaning, cooking) and used the findings to explore how these practices affect energy use. Anthropologists and sociologists consider individual choices to be determined by technological and social systems and for any change in energy use to be the result of a wider social change. This was clearly highlighted in Shove’s [51] text where she outlined and critiqued the pervasive nature and role of technology practitioners and designers in affecting, validating, refining and re-creating consumption norms especially in the home where consumption practices are very much entwined in concepts of cleanliness and comfort.

Electricity demand appears to be deeply rooted in the whole supply chain for electricity services [52, 53] and the social normality of cleanliness, convenience and comfort [51]. Such powerful social norms obviously affect the influence of interventions to change residential energy use behaviour. The attraction of mass population behaviour change has brought some scholars, for example, [3, 11, 14, 38] to the view that a multi-disciplinary approach has the greatest potential for success at the broader level. An integrated approach to the multi-faceted challenge of behaviour change can apply specialist discipline expertise while recognising the issue’s larger and more complex context [11].

### Working with complex systems to develop reality based strategies

As mentioned above, the outcomes of human behaviour and natural systems are often uncertain and complex and require a different approach to one strictly based on logic and structure [46, 47]. Complexity arises where the network of factors affecting the system and its interactions are so involved that it is impossible to track the resultant processes including features such as self-organisation and emergent behaviour [47]. This is the basis of complexity science and system dynamic modelling. People tend to invoke a set of mental models to solve problems that consistently underestimate a problem’s complexity and the interaction of feedback mechanisms [54]. Therefore, formal, structural models for managing complex systems have been developed using complexity science and system dynamic thinking, where reinforcing and balancing continuous feedback loops are a fundamental building block of the system [55–58]. System dynamic modelling is particularly useful for studying interacting elements within complex systems on a broad-scale [58] and as the model is accomplished a theoretical statement is created through incorporating hypotheses about causal connections and the outcomes of their interactions [57]. It is often beneficial to structure the system so that it can be manipulated computationally [58]. This may require feedback to be implemented by sequential repetition of a hierarchical framework representing the system.

Although not widely adopted as theory and practice in management and strategy [47], these models have proved successful in helping to avoid policy resistance and in identifying high-leverage policies for sustained improvement [55] as well as improving outcomes and learning within and about the system [54, 59, 60]. Incorporating ideas from complexity science, system dynamic modelling and human behaviour have been shown to achieve better outcomes in a range of fields [46].

As identified above, demand for electricity is part of a very complex system affected by numerous influences and processes (e.g., environmental, physical-technical, social and economic), either directly related to the consumer or their environment. These influences and processes are complex systems in their own right, which impact on and interact with each other to affect residential electricity demand. Their impact can result in emergent behaviour being exhibited, since intervening in one part of the system can have unintended and quite extreme effects in a quite unrelated part of the system. It is therefore necessary to be able to rigorously assess the inter-relationship and impact of current environmental, social, physical-technical and economic variables on residential electricity demand, to be able to model likely future scenarios and possible outcomes of strategies which might be adopted to ensure electricity supply at peak times. This requires a tool which can model complexity within and between systems and model how changes in one element might flow on to others. Only then, can strategies for peak demand management be developed with reduced risk of unintended negative consequences and the greatest likelihood for success.

**Fig 1:**
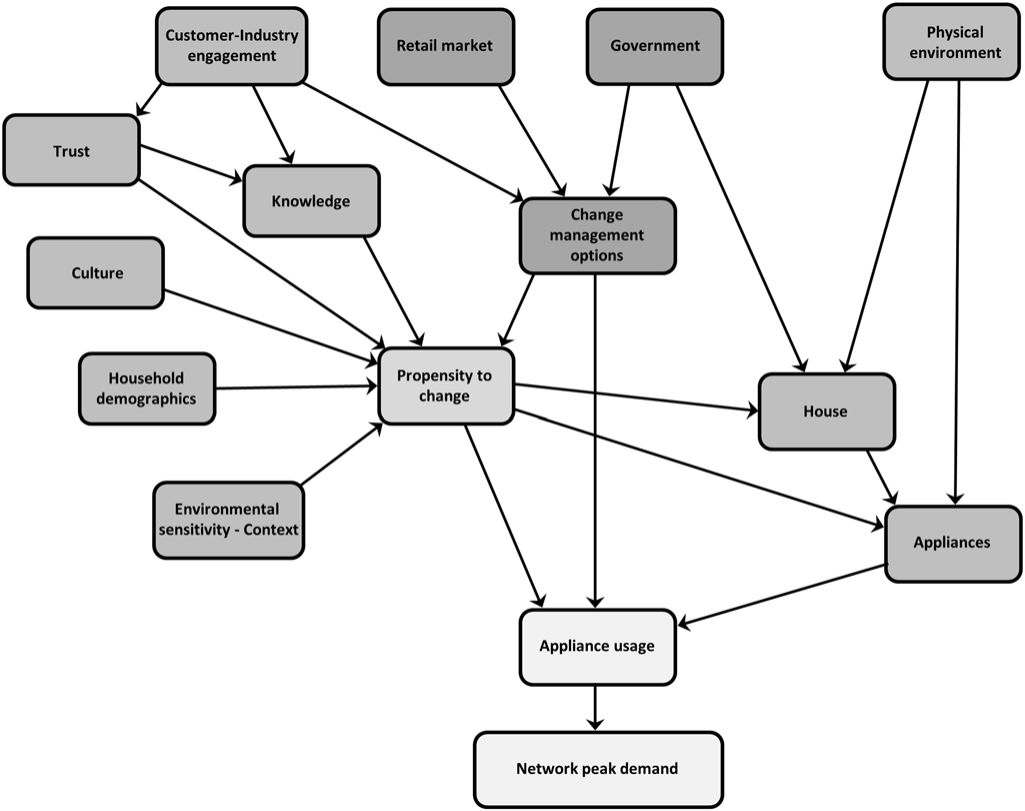
The residential electricity peak demand model.

It has been suggested that any attempt to change electricity use behaviour needs to influence the socio-technical system to be successful [61] and Crosbie (2006) has called for an approach which combines qualitative and quantitative socio-technical research with complex system modelling. This paper discusses the application of a complex systems model designed to incorporate the socio-technical aspects of the system populated with both qualitative and quantitative data specifically to address residential peak electricity demand. Given the level of complexity of energy related behaviour, it is proposed, that residential energy demand could benefit from exploring concepts of human behaviour, system dynamics and complexity science that acknowledge or recognise interacting factors and processes to better understand and research peak energy demand and then be able to use this understanding for designing and evaluating interventions to achieve more reliably successful outcomes of peak demand energy reduction. The framework presented in Fig. 1 has been designed to model the complexity within and between systems and indicate how changes in one element might flow on to others. This is to achieve the dual functions of better understanding energy consumption of different types of households, the factors that impact on residential energy demand at peak times and therefore obtain a better understanding of peak demand behaviour and secondly, as a means with which to design and evaluate interventions as integrated and effective solutions to the problem of residential peak energy demand. As Wilson and Dowlatabadi [14] point out, these are two distinct functions that pull in different directions where completeness and complexity are needed to understand behaviour and where simplicity and parsimony are required for interventions and one may not be readily applicable to the other. The current model is designed to address these two distinct functions.

## Method—Building the Model

### Background

This research was part of a larger study looking at Electricity Demand Side Management: Models, Optimisation and Customer Engagement. The aim was to facilitate identification of critical factors and control points in the complex interactions between technical and social components affecting residential energy demand and in the evaluation of scenarios. The objectives of the initial stages of the project were to bring together disparate knowledge from a wide variety of sources including other research, raw data from consumer focus group research undertaken by the state based and owned utility and to create a ‘conceptual map’ of the social and technical drivers. This paper relates to the creation of the ‘conceptual map’ that would drive and underpin the whole project. No separate ethical approval was obtained for this project as members of the project team (staff from Queensland University of Technology and Ergon Energy) were the only participants involved in the development of the model. The staff involved provided informed consent through the contractual arrangement of the project.

### The Model Building Process

Model building of complex issues requires effective planning and execution sessions that engage key stakeholders and manage any conflict productively [54]. Therefore, the first step in developing our model was to establish an expert committee (key stakeholders) formed by members of the research project team including academic and industry social scientists, engineers, mathematicians and statisticians. The key stakeholders of the project interacted in several group model-building sessions where the issue and the purpose of the project problem of residential peak energy demand were extensively discussed and crafted in dialogue within the group through face to face meetings and through email exchange. These preliminary statements were discussed, changed and finally agreed upon before the first meeting of model development. Initially, our model was based on the integrative models developed by Van Raaij and Verhallen [41] and Keirstead [11]. These models were circulated via email prior to the first meeting and then again in hard copy at the first meeting as a starting point of model development to address the specific problem issue of residential peak energy demand. At the first workshop meeting the expert committee were ‘walked through’ these models and then together the group over several subsequent face to face meetings undertook the process of mapping out our model.

During the process of the face to face workshop meetings, the expert committee were informed by a comprehensive review of empirical research and results of industry led customer research. The subsequent workshop meetings were predominately theoretical sessions for practical trial where the reflections provided led to iterative improvements of early model versions and to the identification of additional data requirements. The ongoing iteration of the model framework was circulated within the group in graphical format. Graphical representation of the evolving model simplified participatory development with stakeholders and information sessions with staff of the industry partner. At each stage the model was assessed by the group to ensure that the system was being accurately represented with causal loop modelling being undertaken on an ongoing basis during the model development. Feedback polarity between the variables was identified and understanding of the causal structure was critical to the continuing development. These sessions helped to frame and test the model as well as preform policy, scenario and consequence analyses. Policy, scenario and consequence testing was undertaken in the form of a ‘what if’ style of analysis. One major benefit of this exercise was the shared understanding of the influences on and of peak energy demand, from a supply and demand perspective and the complexity of their inter-relationship. During the whole process communication and process facilitation was a priority in order to achieve a model that accurately reflected and explained residential consumer peak energy demand.

The modelling method followed standard good modelling practice as identified by Hovmand and colleagues [54] and others [55, 58, 62]. The model evolved in the form of a discourse, in which different key project stakeholders were involved. What kept the discourse going was the model in its various stages built by the group. The process undertaken involved a non-linear sequence from assumptions to review of empirical evidence to hypotheses. The process was iterative and the iterations occurred between the steps of procedure. These actions led to a deeper understanding of the model structure and the causal and inter-relationship between each theme within the model, the system’s structure and consumer behaviour. These tests also helped to refine and adjust the model and strengthen analyses. This whole process established the model’s credibility and nurtured a sense of ownership in the model within the group.

## Results

### The residential electricity peak demand model

**Fig 2:**
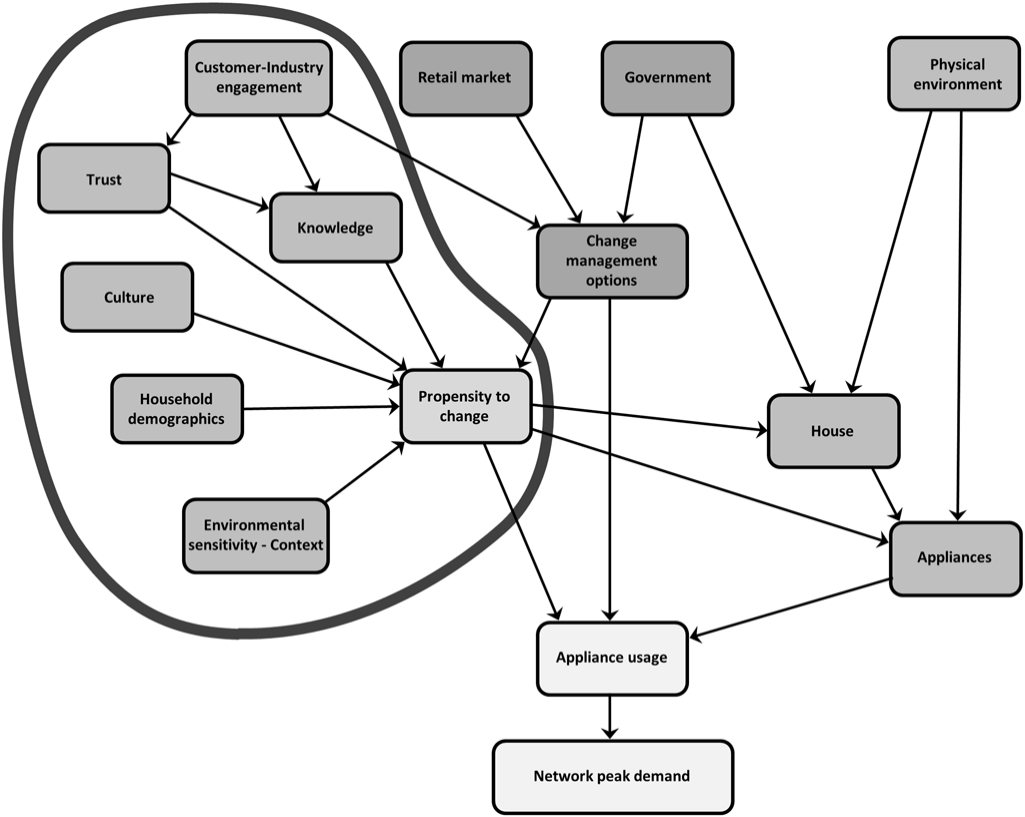
Residential electricity peak demand model with the Propensity to change theme highlighted.

**Fig 3:**
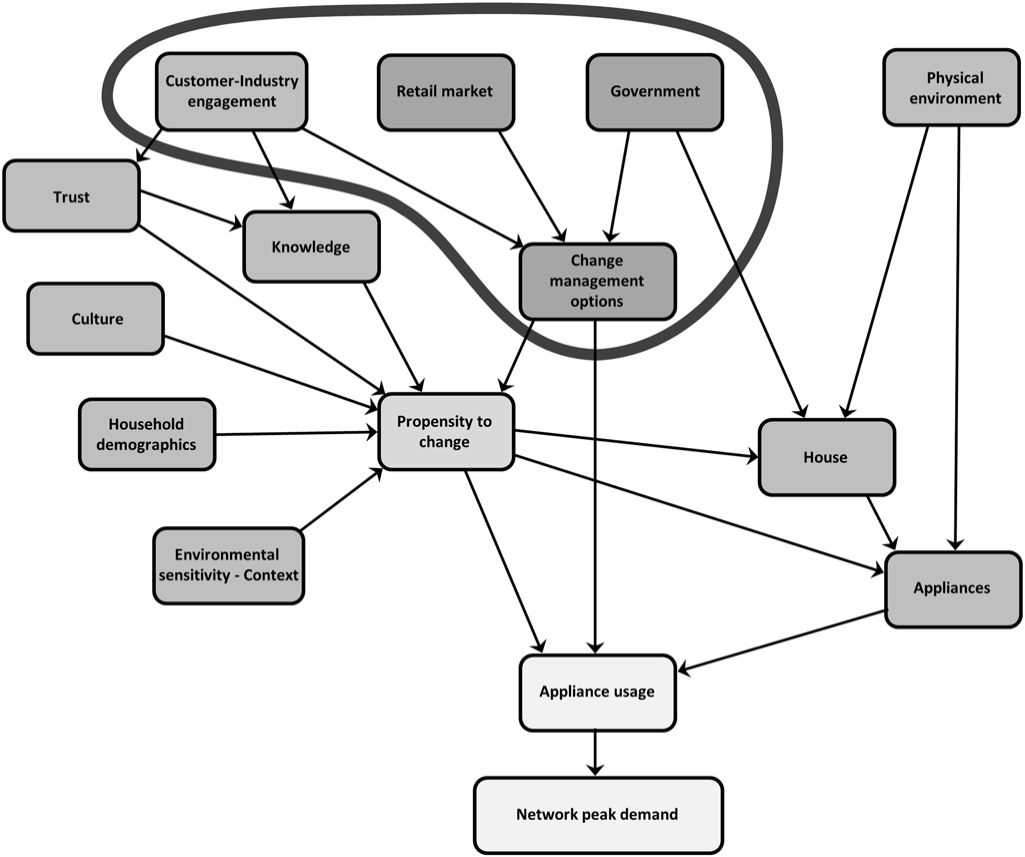
Residential electricity peak demand model with the Change management options theme highlighted.

**Fig 4:**
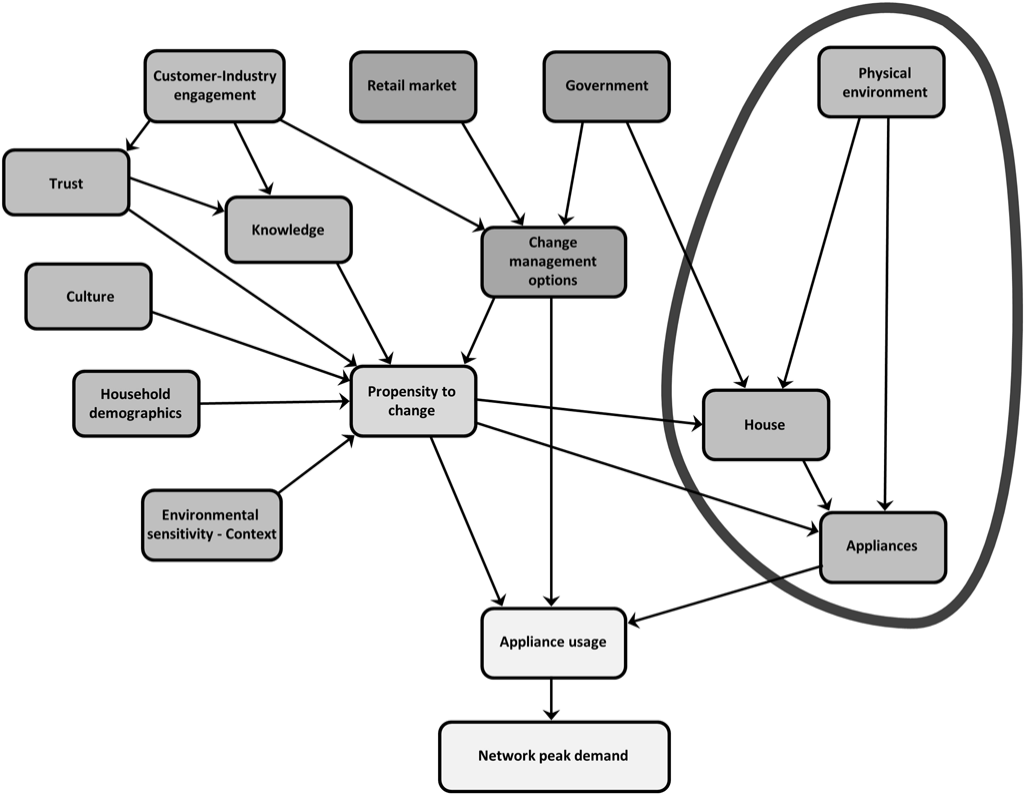
Residential electricity peak demand model with the physical environment theme highlighted.

**Fig 5:**
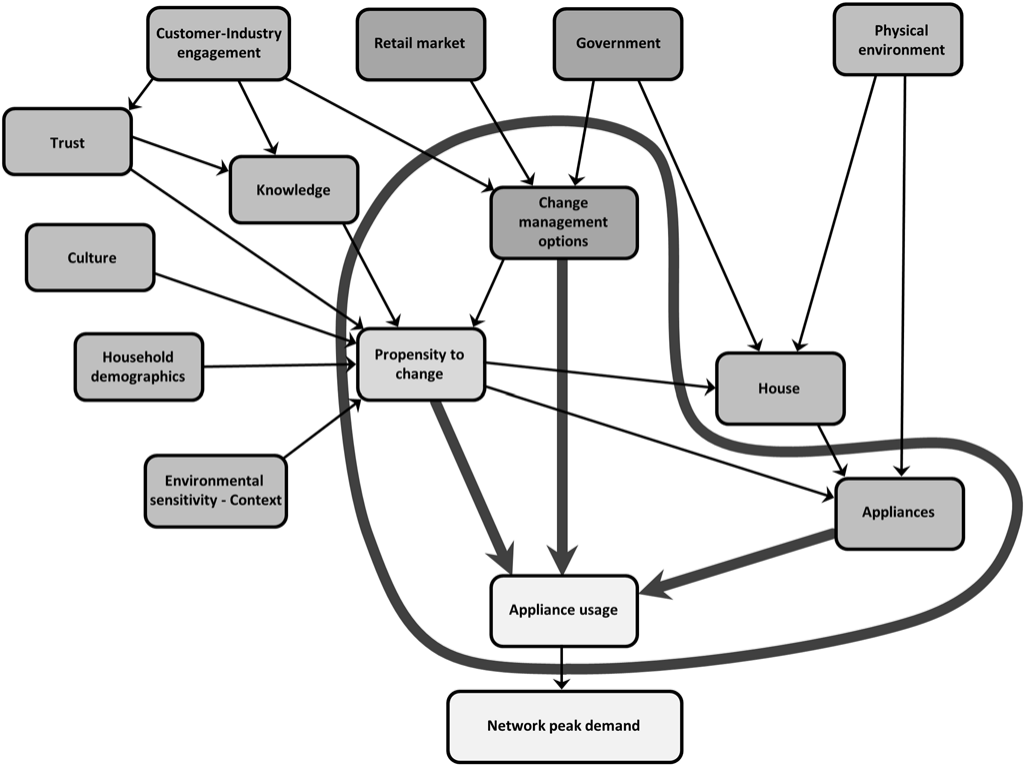
Residential electricity peak demand model with the grouping of appliance usage and network peak demand.

The Residential Electricity Peak Demand model depicted in Fig. 1 has been developed identifying the factors as themes with indicators that lie within each of them. The themes of the model can be described as interlinked components within the core groups of propensity to change (see Fig. 2), change management options (see Fig. 3), appliances (see Fig. 4) and finally the combination of these themes (see Fig. 5) to affect residential peak demand. Feedback is implemented conceptually by time-slicing and sequential repetition. The causal relationships between the themes lead to the model behaviour patterns.

Given the economic and environmental impacts of high levels of electricity consumption, this model attempts to understand how the social, together with the technical and environmental factors interact to affect expansion and contraction in electricity demand during network peaks.

The physical characteristics of the built environment and appliances are an important focus of this model as are the technical, economic and human behavioural aspects of energy consumption. The current model considers expansion or contraction in energy demand as being shaped from an inter-play between all of these aspects/characteristics. Unlike past research, this model does not exaggerate the importance of energy prices and technological solutions at the expensive of social action and non-economic influences.

### Propensity to change—Social theme characteristics

The social characteristics are outlined within the propensity to change grouping of the model and highlighted in Fig. 2. Customer-industry engagement falls within two groupings. The model outlines customer-industry engagement in the hierarchical relationship with trust and knowledge and it is also depicted in the change management grouping. The inter-relationship of customer-industry engagement across both groups is such that it is difficult to isolate it without significant redundancy of message in the discussion of both sections. Therefore, its application will be discussed within this section—the social theme characteristics.

Parag and Darby [63] highlighted the importance of trust in the relationships between residential consumers, the government and electricity suppliers and asserted that the combination of a lack of consumer trust and loyalty in suppliers, a lack of obligation by consumers to reduce their energy demand and price based competition between suppliers (promoted by the government regulator) has been problematic in addressing the issue of energy demand reduction. They suggested Hardin’s concept of trust as ‘encapsulated interest’ on the basis that limited trust does exist between suppliers and consumers as they need one another with the government having the important role of shaping common goals and providing incentives that align some of the supplier and consumer interests [63–65]. A lack of trustworthiness makes it difficult to deliver messages related to values and therefore increase consumer knowledge of energy consumption and peak demand. The public are often wary of politicians’ intentions and mistrust mass media and industry sources of information [66, 67]. Due to this intrinsic suspicion, consumers can be disbelieving of energy saving objectives developed and promoted by government, business and industry [38].

People know little of their energy use related to their behaviour [15] and it has been argued that a deeper knowledge of everyday energy consumption activities makes everyday life more sustainable [68]. It has been suggested that without complete information, consumers are imperfectly rational [69] but that with full information, consumers would maximise utility for money spent and therefore act rationally [70]. However, residential energy consumption is less tangible and requires less active engagement than other forms of consumption, e.g. fueling a car or topping up credit on a phone. In Australia, an un-itemised and non-visual quarterly electricity bill is the only electricity consumption information many consumers receive [71]. The extent of the information provided on these bills has been compared to driving a car without knowing the volume or price of the fuel consumed [72]. This lack of information has become a significant issue in Australia due to residential electricity prices increasing by more than 110 per cent in the last five years with a further projected increase of seven per cent for 2014–2015 [73].

Studies of houses occupied by demographically similar families have reported large (between 200–300%) variations in energy use [74–76]. Type of dwelling, urban/rural location, size, ownership, tenure, attributes of the occupants including the number residing in the residence, their ages, income and occupancy patterns had differing but significant effect on electricity consumption [77–79]. They found a strong correlation between floor area and consumption and while greater floor area is more affordable to higher income households and leads to greater electricity use, the pattern of use is different between income groups with the daily demand profile being 60% larger and the evening profile being 100% larger [77]. Differences among ethnic groups in family size, housing characteristics and appliance holdings certainly influence consumption differences [12, 38, 79] and this model is designed to assign appropriate weights to the various components of consumption.

The effects of contextual factors on environmental sensitivity and growing environmentalism can be linked to opinions about energy [80, 81]. From the 1970s to early 1980s energy and environmental concerns had public attention, this waned in the 1980s and regained interest in the 1990s [82, 83]. However, the public’s conception of the complex connections between environment, energy and policy are not clearly understood [84, 85]. Cultural values such as ‘reducing waste and carbon footprint’, ‘being green’, ‘being independent’ or injunctive norms that somehow indicate what is commonly socially acceptable (or unacceptable) within a certain culture can result in significant energy reduction [38, 86].

The role of habit in energy consumption has often been overlooked by energy researchers [87] even though people do many daily tasks like using electricity according to routines without any or little conscious thought [88]. Cultural practice of people’s routine activities such as bathing, cleaning, cooking establishes the habits of home energy consumption. As mentioned above, residential energy use is variable and changeable and therefore not generalisable across demographic groupings or cultures [88]. Sociocultural norms along with technology affordances, the built and natural environment and infrastructure heavily influence personal and domestic consumption [48]. Appliance specific behaviour appears to vary in ways associated with cultural and lifestyle differences between households [12, 89, 90].

### Change management options

The change management options grouping of the model incorporates technical-economic characteristics. These characteristics are usually set through the retail market or government policy and the effects of energy policy and why policy preferences change over time—before and after policy implementation need to be analysed [87].

Energy conservation and efficiency have been the focus of energy policy of many western governments and utility retail markets since the oil embargos and price spikes of the 1970s. Energy efficiency and energy conservation are often covered together in studies, see for example, [91–95]. In a recent paper, Croucher [96] differentiated between energy efficiency and energy conservation, identifying them as separate but interrelated ways of aiming to reduce electricity consumption. Energy efficiency typically involves reducing the electricity-intensive nature of the production process thus attempting to adjust input requirements for a particular output or consumption decision whilst energy conservation concentrates on decreasing the total amount of goods and services consumed, which then decreases the amount of electricity needed [96]. While the depiction of energy efficiency is complex there have been measurable improvements in the energy efficiency of buildings, residential appliances and equipment and in research funding and development of all energy efficiency technologies with these technologies playing a dominant role in future policy development and implementation [94]. Energy conservation measures, however, are said to “fall foul” to consumer surplus as foregone satisfaction associated with energy conservation is said to increase as consumers engage in more and more energy conservation measures thereby placing limits on the effectiveness of energy conservation programs [96].

Investigating electricity consumption in terms of both price optimisation to industry and consumers and seeking answers other than direct pricing has been the pursuit of economic researchers. The goal has been to assist public policy and regulation in creating energy markets that are beneficial for the community in the long term. Based upon the notion that people attempt to maximise their satisfaction with all given knowledge, economic models seek to understand how energy prices, income, expenditure and taxes affect energy consumption [27, 78, 92, 97, 98]. Rational economic models, however, have failed to predict how consumers will respond to economic incentives to reduce consumption and conserve energy. The difference between actual consumer behaviour and rational economic efficiency has been labelled the ‘energy gap’ [97]. The energy gap is said to be the result of two failures—market and non-market [99]. Market failure is said to occur when there is a lack of information about the full cost of each consumption decision, knowledge about which appliances are the most efficient and when the principal user is not paying for the electricity directly. Non-market failure includes social factors such as preferences for particular cleaning practices or appliances, e.g. using hot water for washing clothes and or choosing incandescent lights over LED or fluorescent. It can also include uncertainty about future energy prices [99]. Economic conceptualisation in explaining and modelling human behaviour and energy use, as Keirstead [11] has previously indicated, is insufficient and yet there has been heavy reliance on economic theories which has misinformed policies and mislead analysis away from social and psychological factors [100].

### Appliances—Natural and built environment characteristics

The importance of acknowledging and accounting for the impact of the physical or natural environment on residential peak energy demand can be linked to evidence of environmental conditions, such as the weather, having a strong effect on appliance use. Analysis of appliance use in Australian homes demonstrated some form of weather sensitivity with the relationship between outdoor weather and individual appliance energy consumption consistently stronger in the cooling season (summer) than the heating season (winter) [101]. In a study undertaken in Northern Ireland, researchers found that average winter consumption exceeded the average summer consumption [77]. The difference in the findings can be attributed to geographical variation with the Hart and de Dear study being undertaken in the southern hemisphere where summers are more severe than winters in terms of residents’ comfort levels. The Yohanis and colleagues’ study was undertaken in the northern hemisphere where residents’ comfort levels are more challenged in winter than summer. In an Indian study where the summers are very hot, it was found that price elasticity was significantly lower in summer rendering future price increase policy on appliance use and hence energy use ineffective in reducing future demand [79]. The current model also requires analysis of the residential built environment and appliances recognising there has been considerable variation in average consumption and load shapes for different appliances and building systems [102]. There is considerable variability in location, style, size, age of housing stock and degree of energy saving features such as insulation with a clear correlation being found between floor area and average annual electricity consumption [77, 103]. There is also substantial variability in number, style, size, age and type of appliances with strong upward demand trends in several areas of household consumption including expanded use of air-conditioning, the purchase of a broader range and greater number of household appliances and increased per capita use of hot water [104]. Purchasing more efficient appliances and changes In the use of appliances can have a significant effect on energy consumption [104]. Renovations and new house efficiencies have been two areas of considerable energy-efficiency policy and research attention [14, 105]. The model allows or encourages analysis of how residential consumer behaviour interacts with the natural and built environments and appliances to influence energy consumption.

## Discussion: Appliance Usage and Network Peak Demand—Bringing It Altogether

This model addresses the heterogeneity of the different end-users with respect to both technical and social components. It incorporates the social components that affect propensity to change, the change management options relating to the retail market and government along with the physical environment of the weather, appliance ownership, built environment floor space and the like. All of this then culminates into assessment of the appliance usage and its effect on network peak demand. Our model illustrates that energy policies, namely on effective peak energy consumption reduction, should focus specific drivers behind each end-use on both technological and social factors in addressing appliance usage and peak demand.

There are multiple, time varying factors that impact electricity demand in Australia’s residential sector and our model has been developed as a means to examine this complexity. Also, the model is comprised of themes that are networked together in a way that captures each theme’s influence, impact or association with other themes in the network. It is possible to see each variable separately as different factors impact on a consumer’s desire or ability to reduce or shift electricity consumption. The number of variables targeted must be responsive to the heterogeneity and complexity of the system. For this reason, we have adopted complexity science and system dynamic theory and modelling to understand the factors that impact on reducing or shifting consumer demand in peak times. The selection of themes and the value that is assigned is made with the aid of research and some reasonably confident assumptions of the system through expert opinion and discussion, thus, demonstrating an integrated, cross-disciplinary approach. By adopting a more multi-disciplinary approach to affect on-going change it gives a more coherent picture of the problem being addressed allowing for robust policy decisions to be made.

To develop this model, internal stakeholders were actively consulted. Through active stakeholder engagement, the model was enhanced by stakeholders’ knowledge and understanding of peak demand management and its dynamics under various conditions. It was a process of collaborative learning which identified and clarified the impacts of solutions to the problem of residential peak demand management which supports decision making and policy development. The process undertaken has deepened our understanding of the connections between the model’s structure and its dynamic behaviour adding substance to intuitions or providing confidence in discounting or discarding them altogether. Obtaining great insight and understanding of the problem appears to be a robust outcome of system dynamics group model building [60, 106]. The effective learning transpires as group participants tackle complex issues and become actively engaged in building the system dynamics group model [62].

The model has been developed through a group model building process as a systems model of the problem situation. It is based on past efforts of proposing models designed to aid our understanding of the effects on residential peak energy demand in a systematic and comprehensive way. There has been the opportunity to source converging evidence of key issues and agreed measures of important variables. The model has been designed to incorporate the socio-technical aspects of the system in order to identify the action required to address the sustainability of electricity supply in the residential sector during times of network peaks.

### Theoretical significance

As discussed above, there have been multiple interventions developed by various disciplines (including technology, economics, psychology and social science) in the last 40 years. Whilst these disciplines have made significant contributions to intervention knowledge individually, there have been calls for a multi-disciplinary approach [11, 14]. This research will make a major contribution as it incorporates a multi-disciplinary focus to the investigation of the factors that impact residential peak electricity demand and influence conservation behaviour or load shifting of electricity demand during peak times.

During the modelling process, theories appeared on a continuing basis as sets of hypotheses that explain the inter-relationship between the dependent variables within the model and how these variables are likely to behave with the introduction of a particular intervention (the independent variable). Theories emerged because they were, in principle, the stronger options chosen. It has been suggested that the basic value of a properly constructed model is that it embodies propositions which can be refuted, has explicit underlying assumptions, operationalises the variables and parameters and undertakes adequate procedures of model validation [55, 106, 107]. We are confident that we address all of these criteria with our model and that we have captured a highly developed system structure that can be used not only to understand the local case that precipitated the model’s development but also for any peak energy demand problem or issue more broadly. We believe that this model generates middle-range theory as it lies between the minor but necessary working hypotheses that evolved during day to day research and the all-inclusive systematic efforts to develop a unified theory that explains all the observed uniformities of social behaviour, social organisation and social change as outlined by Merton in Schwaninger and Grösser [57].

This paper highlights the potential that multilevel and spatial modelling approaches hold for understanding determinants of peak energy demand. Multilevel models separate the variation in an outcome into individual and group or area-level components [108]. The adopted approach was very flexible in terms of accommodating different types of variables and increasing model complexity and the model enables comparison of the influence of household-level co-variates and community and state level contextual effects on electricity peak demand and to assess different strategies to abate peak energy demand. Because of the adopted approach and its design, the model has facility to classify the comparative roles and interactions of, and links between, various disciplines to fully investigate the research problem. It is expected that the model will provide a means for integration of research outcomes from our multi-disciplinary approach. The outcome of interest is the peak demand reduction. It is envisaged that this multi-level modelling approach has potential for understanding the determinants that affect or influence electricity demand in peak periods.

### Practical significance

This model builds on the work of previous frameworks and models developed by Van Raaij and Verhallen [41], Stephenson and colleagues [3]and Keirstead [11]. The current model differs from previous examples in that it has been specifically designed to tackle peak energy demand. Another point of difference is the model’s development with expert opinion informed by experience and published research. The iterative process led to a deeper understanding of the connections between the model’s structure and its dynamic nature resulting in greater insight and understanding of the peak energy demand problem for the utility and opportunities for its solution. The Residential Electricity Peak Demand Model allows for variation and selection where options are created and tested providing the opportunity to see how particular interventions might work. An intervention is a complex system in itself, consisting of a number of elements together and, in interaction, producing the outcome of the intervention. The Residential Electricity Peak Demand Model considers the issue of behaviour and its numerous manifestations by allowing for the effects of exchanges between culture and energy practices, socio-demographics, trust, knowledge, environmental sensitivity and demographics. The current model accounts for a variety of influences of behaviour, through the modelling of the interactions between the core nodes of behaviour and more extensive technical, social and structural effects. The model is fluid in nature accommodating variation and evolution rather than viewing decisions as inevitable. It accounts for the broader structural powers of the economy, environment and society without assigning total governance of these powers over residential consumer behaviour. The model allows for exploration of the different nodes to identify opportunity and likely impact for any intervention to achieve behavioural change in reducing peak electricity demand.

This research will facilitate future development of conservation and peak demand reduction innovation and policy tools to improve outcomes for diverse stakeholders, including long-term energy security, avoiding over-capitalisation in the electricity network and lower electricity bills for consumers. If conservation and peak demand reduction interventions prove successful across all consumer groups, the Queensland Government forecasts that by 2020, a conservation and peak demand reduction program has the potential to deliver a reduction in Queensland peak electricity demand of over 1,100 megawatts and $4 billion in electricity infrastructure capital expenditure, energy savings of over 22,220 gigawatt hours, greenhouse gas emissions savings of over 23,200 kilotonnes, and water savings from reduced electricity generation of over 42,200 megalitres [4].

## Conclusion

The most important goal of this study was for system improvement in dealing with residential peak energy demand. The factors that affect residential peak electricity demand are complex and require a dynamic model to optimise the diagnosis and strategy development to manage it. This paper details a dynamic object-oriented model to formalise the complex dynamic system which is residential peak demand. The model has undergone many iterations of evaluation to determine its general reliability and the merit and impact of each of the factors incorporated within the model. The real value of this model is in utilising the a priori knowledge of previous implementations detailed in literature and the expert knowledge of the internal stakeholders who made significant contribution during its development.

The model building process was helpful in creating awareness, understanding and insight into the complexity of residential peak energy demand and in being able to identify and integrate the social, technical and change management option themes and their impact on appliance usage and residential energy demand at peak times. Discerning which methods work best for particular problems is an area for future research but one that will require clear understanding of the complexity of the problem. This paper makes a contribution to this field by outlining an integrated approach for addressing residential peak energy demand.
